# Genetic structure, diversity, and allelic richness in composite collection and reference set in chickpea (*Cicer arietinum *L.)

**DOI:** 10.1186/1471-2229-8-106

**Published:** 2008-10-16

**Authors:** Hari D Upadhyaya, Sangam L Dwivedi, Michael Baum, Rajeev K Varshney, Sripada M Udupa, Cholenahalli LL Gowda, David Hoisington, Sube Singh

**Affiliations:** 1 International Crops Research Institute for the Semi-Arid Tropics (ICRISAT), Patancheru PO, 502324, AP, India; 2 International Center for Agricultural Research in the Dry Areas (ICARDA), PO Box 5466, Aleppo, Syrian Arab Republic

## Abstract

**Background:**

Plant genetic resources (PGR) are the basic raw materials for future genetic progress and an insurance against unforeseen threats to agricultural production. An extensive characterization of PGR provides an opportunity to dissect structure, mine allelic variations, and identify diverse accessions for crop improvement. The Generation Challenge Program  conceptualized the development of "composite collections" and extraction of "reference sets" from these for more efficient tapping of global crop-related genetic resources. In this study, we report the genetic structure, diversity and allelic richness in a composite collection of chickpea using SSR markers, and formation of a reference set of 300 accessions.

**Results:**

The 48 SSR markers detected 1683 alleles in 2915 accessions, of which, 935 were considered rare, 720 common and 28 most frequent. The alleles per locus ranged from 14 to 67, averaged 35, and the polymorphic information content was from 0.467 to 0.974, averaged 0.854. Marker polymorphism varied between groups of accessions in the composite collection and reference set. A number of group-specific alleles were detected: 104 in Kabuli, 297 in desi, and 69 in wild *Cicer*; 114 each in Mediterranean and West Asia (WA), 117 in South and South East Asia (SSEA), and 10 in African region accessions. Desi and kabuli shared 436 alleles, while wild *Cicer *shared 17 and 16 alleles with desi and kabuli, respectively. The accessions from SSEA and WA shared 74 alleles, while those from Mediterranean 38 and 33 alleles with WA and SSEA, respectively. Desi chickpea contained a higher proportion of rare alleles (53%) than kabuli (46%), while wild *Cicer *accessions were devoid of rare alleles. A genotype-based reference set captured 1315 (78%) of the 1683 composite collection alleles of which 463 were rare, 826 common, and 26 the most frequent alleles. The neighbour-joining tree diagram of this reference set represents diversity from all directions of the tree diagram of the composite collection.

**Conclusion:**

The genotype-based reference set, reported here, is an ideal set of germplasm for allele mining, association genetics, mapping and cloning gene(s), and in applied breeding for the development of broad-based elite breeding lines/cultivars with superior yield and enhanced adaptation to diverse environments.

## Background

Chickpea (*Cicer arietinum *L.) is the 4^th ^most important grain-legume crop after soybean, bean, and pea, but contributes only 3.1% to the world grain legumes production (based on 2001 to 2006 average production of 266.5 million tons of soybean, beans, peas, chickpea, broad beans, cowpea, lentil, and pigeonpea) , assessed on 27^th ^January 2008). Worldwide chickpea production in 2006 was 8.24 million tonnes (Mt) from an area of 9.4 million ha, and average productivity of 0.77 t ha^-1^. Asia contributes 89.4% (7.36 Mt), Africa 3.9% (0.32 Mt), North and Central America 4.9% (0.40 Mt), Oceania 1.3% (0.11 Mt) and Europe 0.5% (0.04 Mt) to world chickpea production. Over 50 countries grow chickpea; however, India, Turkey, Pakistan, Iran, Canada, Myanmar, Mexico, Ethiopia, and Australia together contribute 93.1% of the global chickpea production. Although North and Central America and Oceania together contribute only 6.2% of the world chickpea production, these regions have the highest recorded chickpea productivity (1.09 t ha^-1 ^to 1.34 t ha^-1^). In contrast, Asia and Africa show the lowest productivity (0.75 t ha^-1 ^to 0.79 t ha^-1^) while contributing 93.3% of the world chickpea production. Several biotic and abiotic stresses [1,2], its narrow genetic base [3] probably as a consequence of its monophyletic descendent from its wild progenitor *C. reticulatum *in the Fertile Crescent [4] and lack of adapted varieties contribute to fluctuations in chickpea yield.

Chickpea is a self-pollinated crop, with 2n = 2x = 16 chromosomes and genome size of 732 Mb [5]. The two distinct forms of cultivated chickpeas are desi types (small seeds, angular shape, and coloured seeds with a high percentage of fibre) and kabuli types (large seeds, ram-head shape, beige coloured seeds with a low percentage of fibre). A third type, designated as pea-shaped, is characterized by medium to small seed size, and cream coloured seeds. The desi types are primarily grown in South Asia, while kabuli types mainly in Mediterranean region. Chickpea is the good source of carbohydrates and proteins, together contributing about 80% of the total seed dry weight. The chickpea grains are rich in minerals and vitamins, and also forms a good source of livestock feed.

Vast collections of chickpea germplasm are maintained by two CGIAR (Consultative Group on International Agricultural Research) centers: the International Crops Research Institute for the Semi-Arid Tropics (ICRISAT), Patancheru, India and the International Center for Agricultural Research in the Dry Areas (ICARDA), Aleppo, Syrian Arab Republic. The former maintains 17,258 accessions (135 wild and 17,123 cultivated) while the latter 12,647 accessions (304 wild and 12,343 cultivated). In spite of such an impressive number of germplasm accessions available in the genebanks, there has been very limited use of these accessions in genetic enhancement of chickpea. For example, the chickpea breeders in ICRISAT used only 83 germplasm lines during the period from 1978 to 2004 in comparison to the use of 480 breeding lines for the development of 3430 advanced varieties. A similar situation was noted at ICARDA, wherein the chickpea breeders during the same period used approximately 250 germplasm lines in their crosses, compared to approximately 600 breeding lines in generating breeding materials, from which 31 cultivars were released. India is the largest producer of chickpea and has a very strong chickpea improvement program, which released 126 cultivars during 1967 to 2003. The pedigree analysis of the 86 released cultivars, developed through hybridization, indicated that though 95 ancestors were involved in their development, only 10 accessions contributed to 35% of the genetic base [3].

The development of core and mini-core collections has been suggested as a gateway to the utilization of genetic diversity in crop improvement [6,7]. In chickpea, core and mini core subsets have been reported [7,8]. More recently, a composite collection of 3000 accessions have been developed [9] which included the 1956 accessions of the ICRISAT core collection [8], 709 ICARDA cultivated genebank accessions, 39 advanced breeding lines and cultivars and 241 trait-specific accessions (resistant/tolerant to biotic and abiotic stresses, early maturity, multi-seeded pods, double podded, large-seed size, high seed protein, nodulation and responsiveness to high input conditions) [9], and 20 wild *Cicer *species (*C. echinospermum *and *C. reticulatum*) accessions. Both *C. echinospermum *and *C. reticulatum *are cross compatible with cultivated chickpea (*C. arietinum*), and reported resistant to several pests (cyst nematode, leaf miner and bruchid) and diseases (fusarium, ascochyta blight and phytophthora), tolerance to cold, and high seed protein content in *C. reticulatum *[10]. This composite collection consists of 80% landraces, 9% advanced breeding lines, 2% cultivars, 1% wild relatives, and 8% for which precise status is unknown. Geographically, 39% of the composite collection originates from South and South-East Asia (SSEA), 25% from West Asia (WA), and 22% from the Mediterranean region. Africa and Americas each contribute 5% of the collection. This composite collection thus represents a wide spectrum of genetic diversity captured from the entire collection of chickpea germplasm preserved in ICRISAT and ICARDA genebanks.

Knowledge and management of the genetic diversity in cultivated and wild relatives are critical for any crop improvement programs. Hybridization, seed protein electrophoresis and isozyme analysis, prior to the discovery of PCR-based markers, were used to establish genetic relationships among *Cicer *species [11,12]. Subsequently, markers such as random amplified polymorphic DNA (RAPD), restriction fragment length polymorphism (RFLP), amplified fragment length polymorphism (AFLP), inter simple sequence repeat (ISSR), and simple sequence repeat (SSR) (also known as microsatellite) were used to study the genetic diversity and species relationships in chickpea; with most of these studies reporting abundant diversity in wild *Cicer *but limited variation in cultivated chickpea [13-18]. Efforts were directed towards the discovery and characterization of large number of SSR markers in chickpea [19-23]. Limited studies on SSR-based genetic diversity revealed sufficient polymorphisms in chickpea that led to the construction of genetic linkage maps and identification of quantitative trait loci (QTL) associated with few traits of significant agricultural importance [24-31].

In this article, we analyze the genetic structure, diversity and allelic richness in a composite collection, using SSR markers, and report the formation of a genotype-based reference set of 300 accessions for diverse applications in chickpea genomics and breeding.

## Results

### Allelic richness and diversity in composite collection

The forty-eight SSR markers detected a total of 1683 alleles in 2915 chickpea accessions. The number of alleles per locus ranged from 14 (NCPGR4) to 67 (TA2), with an average of 35 alleles per locus (Table 1). The polymorphic information content (PIC) values ranged from 0.467 (CaSTMS21) to 0.974 (TA176), with an average of 0.854. Most of the markers, except for CaSTMS21, NCPGR4, NCPGR6, NCPGR7, NCPGR19, and TS84, were highly polymorphic. Gene diversity is defined as the probability that two randomly chosen alleles from the population are different. It varied from 0.533 (CaSTMS21) to 0.974 (TA176), with an average of 0.869. A very low level of heterozygosity (%) was detected in the investigated materials, 0.00% to 3.23%, with an average of 0.80%. Fifteen SSR loci detected no heterozygosity while nineteen, six, seven, and one loci, respectively, detected < 1%, < 2%, < 3%, and < 4% heterozygosity in 2915 accessions. Correlation analysis revealed that allele size range was significantly associated with alleles per locus (r = 0.698, P < 0.01) and gene diversity (r = 0.496, P < 0.01); alleles per locus with gene diversity (r = 0.687, P < 0.01); common alleles with allele range size (r = 0.565, P < 0.01), alleles per locus (r = 0.780, P < 0.01), and gene diversity (r = 0.818, P < 0.01); rare alleles with allele range size (r = 0.573, P < 0.01), alleles per locus (r = 0.844, P < 0.01), and gene diversity (r = 0.358, P < 0.05). Significant and positive relationships were observed between allele size range and the amount of variation at SSR loci (as measured by alleles per locus and gene diversity) indicate that SSR loci with large allele range (resulting from large number of SSR units) show greater variation, and agree with the idea that replication slippage plays an important role in the generation of new alleles at SSR loci [31-33].

**Table 1:** Allelic composition, polymorphic information content (PIC), gene diversity, and heterozygosity (%) of the 48 SSR loci in composite collection (2915 accessions) of chickpea

**Marker**	**Allelic composition**					**PIC**	**Gene diversity**	**Average heterozygosity (%)**
	**Allelic richness**	**Size range (bp)**	**Rare allele (1%)**	**Common allele (1- ≤ 20%)**	**Most frequent allele (> 20%)**			
CaSTMS2	29	114	10	19	0	0.929	0.933	2.43
CaSTMS15	29	159	13	16	0	0.904	0.911	1.85
CaSTMS21	20	60	16	2	2	0.467	0.533	0.17
NCPGR4	14	52	10	3	1	0.605	0.649	0.21
NCPGR6	21	148	18	1	2	0.559	0.627	0.11
NCPGR7	15	42	11	2	2	0.549	0.619	0.11
NCPGR12	27	58	15	10	2	0.813	0.832	0.14
NCPGR19	26	176	20	4	2	0.591	0.651	0.11
TA2	67	158	47	20	0	0.948	0.950	0.00
TA3	29	100	21	5	3	0.726	0.764	0.00
TA5	43	138	30	13	0	0.909	0.915	0.00
TA8	33	106	14	19	0	0.913	0.919	0.00
TA11	29	138	17	12	0	0.871	0.882	0.00
TA14	41	144	23	18	0	0.904	0.911	0.80
TA21	42	147	21	21	0	0.938	0.941	0.62
TA22	50	213	17	33	0	0.962	0.963	0.40
TA27	32	114	19	13	0	0.891	0.899	0.75
TA42	46	150	27	19	0	0.936	0.939	0.00
TA46	24	69	11	11	2	0.843	0.858	0.34
TA64	36	111	15	21	0	0.942	0.945	3.23
TA71	41	138	25	16	0	0.917	0.922	0.85
TA72	45	198	33	12	0	0.874	0.885	0.46
TA76s	34	165	24	9	1	0.813	0.834	2.37
TA78	39	167	21	18	0	0.905	0.911	0.00
TA80	35	127	16	19	0	0.927	0.931	0.00
TA96	48	163	29	19	0	0.918	0.923	0.00
TA113	19	87	8	10	1	0.851	0.863	1.63
TA116	33	117	22	9	2	0.838	0.853	0.89
TA117	36	150	18	18	0	0.929	0.933	0.62
TA118	43	147	18	25	0	0.949	0.951	1.55
TA130	23	69	10	12	1	0.821	0.835	1.19
TA135	21	96	8	12	1	0.850	0.863	2.70
TA142	23	84	14	8	1	0.758	0.784	0.57
TA144	56	108	37	19	0	0.931	0.934	0.00
TA176	64	219	25	39	0	0.974	0.974	0.00
TA194	29	204	17	11	1	0.866	0.876	0.00
TA200	39	129	23	16	0	0.916	0.922	1.71
TA203	56	134	27	29	0	0.963	0.965	0.00
TA206	32	99	18	14	0	0.898	0.906	0.71
TAA58	45	144	21	24	0	0.955	0.957	1.33
TAASH	39	132	20	19	0	0.930	0.934	0.70
TR1	62	191	45	17	0	0.929	0.933	0.00
TR7	26	81	8	18	0	0.891	0.899	2.44
TR29	32	132	15	17	0	0.915	0.921	2.08
TR31	16	69	7	8	1	0.843	0.859	2.66
TR43	53	225	26	27	0	0.956	0.958	2.45
TS45	26	140	17	8	1	0.842	0.858	0.00
TS84	15	69	8	5	2	0.596	0.656	0.32
Total	1683		935	720	28			
Mean	35		19.5	15.0	0.58	0.854	0.869	0.80
Range	14–67	42–225	7–47	2–39	0–3	0.467–0.974	0.533–0.975	0.00–3.23

Of the 1683 alleles detected in the composite collection, 935 were rare, 720 common, and 28 most frequent alleles (Table 1). Rare and common alleles were detected at all the 48 SSR loci, the former ranged from 7 (TR31) to 47 (TA2) with an average of 19.5 rare alleles per locus while the latter from 1 (NCGPR6) to 39 (TA176) with an average of 15 common alleles per locus. In contrast, only 18 SSR loci detected 1 to 3 most frequent alleles in the composite collection. Average allele range size of the markers with trinucleotide repeat motifs was greater (135 bp) than those either with dinucleotide (89 bp) or compound (131 bp) repeat motifs markers.

### Heterozygosity in germplasm accessions

Chickpea is a self pollinated crop. Moreover, in this study, a single plant from each accession was harvested and parts of the seeds obtained from such plants were sown in greenhouse to raise seedlings for DNA extraction. Extreme care was taken to avoid inadvertent seed mixtures. In spite of this, allelic heterozygosity was detected in chickpea accessions that ranged from one to 22 loci in 601 accessions (20.6%): one locus heterozygous in 385, two loci in 116, three loci in 47, four loci in 25, five loci in 6, six loci in 7, seven and nine loci each in 3, eight loci in 4, ten loci in 2, and 11, 19, and 22 loci each in 1 accessions (data not presented). In the remaining 2314 accessions, these markers detected no heterozygosity. A large collection of landraces was involved in this study and it is possible that these accessions still possess some residual heterozygosity at least at some SSR loci reported. A landrace is defined as an autochthonous (primitive) variety with a high capacity to tolerate biotic and abiotic stresses, resulting in high yield stability and an intermediate yield level under a low input agricultural system [34]. The heterozygosity observed at some of the loci could also be due to high mutational rate and mutational bias at SSR loci [35]. The loci with large number of repeat units (SSR units) tend to show high mutational rate [35]. As a result, any mutations in any one of the alleles may create a heterozygous condition. Many of the loci which displayed heterozygous status have a large number of SSR units.

Wild *Cicer *accessions as a group were more heterozygous (10.74%) than cultivated forms (0.49% to 1.14%). Mediterranean accessions were more heterozygous (1.51%) than the accessions from rest of the geographic regions (0.34% to 1.19%) (Table 2).

**Table 2:** Molecular diversity based on biological and geographical groupings of the chickpea composite collection (48 SSR loci data on 2915 accessions)

**Group**	**# accessions**	**Allelic composition**			**Unique allele**	**Gene diversity**	**Average heterozygosity (%)**
		**Allelic richness**	**Rare allele (1%)**	**Common allele (1%)**			
Biological							
Kabuli	1167	1288 (27)	597	691	104	0.845 (0.254–0.964)	1.14
Desi	1668	1481 (31)	781	700	297	0.846 (0.417–0.974)	0.52
Pea-shaped	70	670 (14)	7	663	4	0.874 (0.484–0.955)	0.49
Wild *Cicer *species	10	341 (7)	0	341	69	0.791 (0.444–0.890)	10.74
Geographical							
Africa	150	832 (17)	199	633	10	0.801 (0.301–0.949)	0.42
CIS	44	557 (12)	0	557	1	0.795 (0.376–0.929)	1.19
Europe	65	647 (13)	5	642	2	0.804 (0.175–0.936)	0.78
Mediterranean	619	1241 (26)	573	668	114	0.832 (0.260–0.965)	1.51
North and central America (NCA)	94	719 (15)	0	719	6	0.829 (0.466–0.948)	0.34
South America (SA)	49	524 (11)	0	524	3	0.772 (0.119–0.941)	1.08
South and southeast Asia (SSEA)	1138	1322 (28)	678	644	117	0.821 (0.347–0.969)	0.55
Unknown region	34	583 (12)	0	583	14	0.850 (0.545–0.939)	0.31
West Asia (WA)	720	1318 (27)	535	783	114	0.866 (0.478–0.967)	0.72

^+ ^Average number of alleles

### Biological and geographical diversity in the composite collection

Biologically, the 2915 accessions could be grouped into desi, kabuli and pea-shaped, among the cultivated chickpea types, and wild *Cicer *accessions into a separate group while geographically they can be assigned to eight geographical regions, with another group of accessions of unknown origin. Though kabuli and desi showed similar mean gene diversity, the kabuli's as a group were genetically more diverse (high range in gene diversity) than desi's (Table 2). Interestingly, accessions from South America, Europe and Mediterranean regions were genetically more diverse (high range in mean gene diversity) than those from other regions.

This study detected many rare, common, and unique alleles within each group (Table 2). In the cultivated group, desi's contained the largest number of unique alleles (297) followed by kabuli (104) and pea-shaped (4). Sixty-nine unique alleles differentiated wild *Cicer *accessions from the cultivated chickpea germplasm. Mediterranean and WA region accessions each have 114 unique alleles while SSEA accessions 117 unique alleles. Accessions from Africa contained 10 unique alleles. Of the 1683 alleles detected in the composite collection, desi and kabuli germplasm shared the largest number of alleles (436) while wild *Cicer *shared only 17 and 16 alleles with desi and kabuli accessions, respectively. Pea-shaped type shared 7 alleles with desi and 8 alleles with kabuli. The accessions from SSEA and WA shared 74 alleles while those from Mediterranean shared 38 and 33 unique alleles with WA and SSEA, respectively. African germplasm shared more alleles with SSEA (11) than those from Mediterranean (3) and WA (5). Desi's contained a higher proportion of rare alleles (53%) than kabuli's (46%), while wild *Cicer *accessions were devoid of rare alleles. The frequency of common alleles between desi and kabuli types ranged from 47% to 54%, while pea-shaped type had 99% common alleles. Accessions from Africa had more common alleles (76%) than those from WA (59%), Mediterranean (54%), and SSEA (49%). A very high proportion of common alleles (99–100%) found in Commonwealth of Independent States (CIS), European, North Central America (NCA) and South America (SA) accessions probably revealed homogeneity in the genetic materials from these regions. These are the regions that also detected a very low number of unique and rare alleles.

Several differences were detected in allelic richness in the composite collection. Desi and kabuli types possess greater average number of alleles (27–31) than those from pea-shaped and wild *Cicer *(7–14), with more alleles in desi than kabuli (31 compared to 27). African accessions had less alleles than those from the Mediterranean, SSEA, and WA (26–28 compared to 17 in Africa) (Table 2). The average allele size range in desi and kabuli types differ by 12 bp while pea-shaped differed from both the types by 47–59 bp (see additional file 1). Interestingly, Mediterranean and SSEA regions accessions had no difference in mean allele-size range (103.5 to 104.8 bp). The African accessions, in contrast, differ by 36–37 bp from Mediterranean and SSEA region accessions. The WA region accessions differ by 3–4 bp from Mediterranean and SSEA.

### Variation in polymorphic information content (PIC) in composite collection and reference set

Several differences were detected in marker polymorphism (PIC). Lower PIC values for most of the markers were found for the reference set than for the composite collection. However, a few markers were more polymorphic in the reference set than in the composite collection. For example, NCPGR6, NCPGR7, NCPGR19, and TA142 in desi type; CaSTMS21, NCPGR4, NCPGR7, TA3, and TS84 in kabuli types; NCPGR6 and NCPGR19 in pea-shaped types were more polymorphic in reference set (see additional file 2). Region-specific differences in marker polymorphism were also detected: NCPGR4, NCPGR6, NCPGR7, NCPGR12, NCPGR19, and TA142 were more polymorphic in the reference set accessions included from African and Mediterranean regions while none of these markers, except for NCPGR6, were more polymorphic in SSEA and WA region accessions included in the reference set (see additional file 3).

### Genetic structure of the composite collection and a reference set

**Figure 1:**
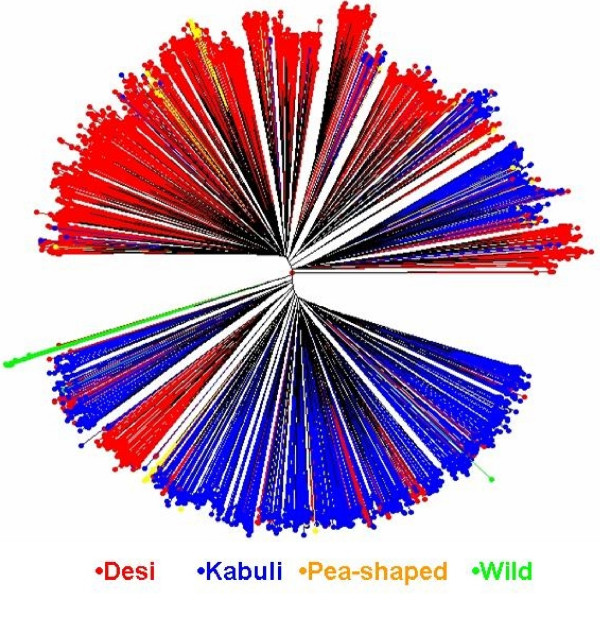
Unweighted neighbor-joining tree based on the simple matching dissimilarity matrix of 48 SSR markers diagram genotyped across the chickpea composite collection (2915 accessions).

Neighbour-joining tree based on simple matching dissimilarity matrix between 2915 accessions of the composite collection highlighted two major groups, broadly representing kabuli and desi types (Figure 1). Clearly, three subgroups could be seen in kabuli while four subgroups in desi types. Further, a group of kabuli accessions clustered with desi types while a group of desi accessions formed a distinct subgroup within the kabuli types. No specific grouping was observed for the pea-shaped accessions, which dispersed in both the groups. Wild *Cicer *accessions clustered with kabuli types; however, *C. reticulatum *accessions (7) formed a distinct cluster separating those belonging to *C. echinospermum *(3 accessions). Both belong to the primary gene pool and are cross compatible with *C. arietinum*, the cultivated chickpea.

**Figure 2:**
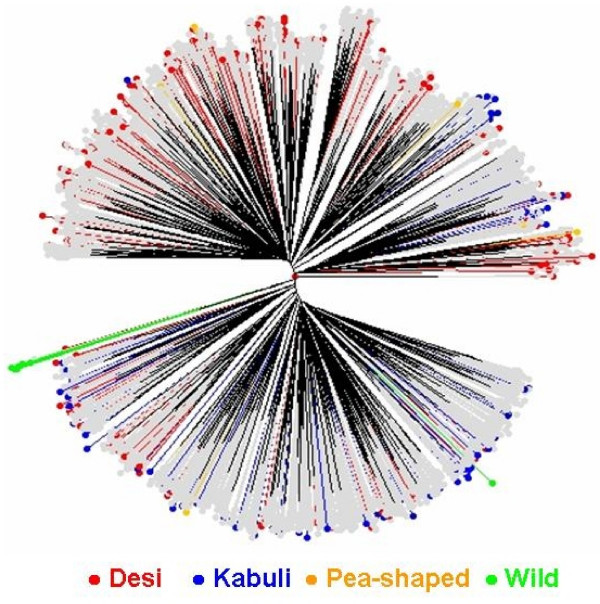
Unweighted neighbor-joining tree based on the simple matching dissimilarity matrix of 48 SSR markers across the chickpea composite collection with proposed reference set (300) accessions identified in red (desi), blue (kabuli), yellow (pea- shaped), and green (wild *Cicer*).

A reference set of 300 accessions (see additional file 4) was formed that captured 1315 of the 1683 (78%) alleles detected in the composite collection of 2915 accessions. The number of alleles per locus ranged from 8 (NCPGR 4 and NCPGR 7) to 56 (TA176), and averaged 27 alleles per locus. This reference set contained 463 rare and 826 common alleles. Rare alleles ranged from 2–20, averaged 9.6 alleles per locus, while common alleles ranged from 0 to 41, averaged 17 alleles per locus. Twenty-six of the 28 most frequent alleles of the composite collection were also detected in this reference set. The gene diversity varied from 0.540 (CaSTMS21) to 0.987 (TA5), averaged 0.881 per locus. Neighbour-joining tree diagram of this reference set (Figure 2) represented diversity from all directions of the tree diagram of the composite collection (Figure 1). Biologically, this reference set consists of 267 landraces, 13 advanced lines and cultivars, 7 wild *Cicer *accessions, and 13 accessions with unknown biological status. Geographically it consists of accessions from Asia (198), Africa (21), Europe (3), Mediterranean (56), Americas (10), CIS (6), and 6 accessions with unknown geographical origin. When accessions classified based on seed types, it has 197 desi, 86 kabuli, and 10 pea-shaped accessions among cultivated types and 7 wild *Cicer *accessions (*C. reticulatum *and *C. echinospermum*).

### Rare, common, and most frequent alleles in composite collection and the reference set

The allelic composition revealed the predominance of rare and common alleles while the most frequent alleles are represented by ≤ 2% of the total number of alleles detected in the composite collection and reference set (Table 3). However, the representation of common alleles in the reference set increased by 14.7% while the rare and most frequent alleles decreased by 50.5% and 7.1%, respectively. A large number of these alleles represented both in the composite collection and reference set: 417, 650, and 22 rare, common, and most frequent alleles, respectively, in composite and reference set, though in varying frequency (data not presented).

**Table 3:** Number of rare, common, and most frequent alleles detected in composite collection and reference set of chickpea

**Allele type**	**Composite collection**	**Reference set**	**Alleles representing in composite collection and reference set**
Rare allele	935 (55.5)	463 (35.2)	417 (38.3)
Common allele	720 (42.8)	826 (62.8)	650 (59.7)
Most frequent allele	28 (1.7)	26 (2.0)	22 (2.0)
Total	1683	1315	1089

Rare allele < 1%, common allele ≥ 1% – ≤ 20%, most frequent allele >20%; figures in parenthesis represents proportion of alleles within composite collection and reference set

## Discussion

Crop genetic resources and the diversity present in them provide an assurance for future genetic progress and an insurance against unforeseen threats to agricultural production. Thus, genetic diversity is of utmost importance to increased yield, enhanced resistance to pests and diseases, and improved grain quality (both grain and stover). Chickpea, like other legumes, has a narrow genetic base in spite of the large collection of germplasm and globally active genetic enhancement program. Knowledge and management of the genetic diversity are critical for any crop improvement programs. Past efforts led to believe that low molecular variation exists in cultivated chickpea; however, this conclusion is based on limited number of germplasm and markers involved in these studies. With the discovery of large numbers of genomic SSR markers, it is now possible to conduct extensive molecular diversity in chickpea for identifying genetically diverse germplasm with beneficial traits for use in crop improvement programs [36]. Towards this end, a composite collection of 3000 accessions [9] has been developed, sampling wide geographical and biological diversity from over 29,000 chickpea accessions conserved in genebanks in ICRISAT and ICARDA, the two CGIAR centers having chickpea improvement programs. In this study, we have molecularly profiled this composite collection using 48 SSR markers. This is the largest and most extensive molecular dataset generated in chickpea, which detected 1683 alleles with high gene diversity, and large number of rare, common, and unique alleles. This study also detected a highly significant (P < 0.01) positive correlation between alleles per locus and gene diversity, allele size with alleles per locus and gene diversity, and common and rare alleles with allele size, alleles per locus and gene diversity. However, variable and inconsistent relationship between the number of repeat unit length and SSR polymorphism has been reported in several legumes including chickpea [37]. Information available on these alleles present in different germplasm lines will be very useful for developing the mapping populations for genome analysis as well as in applied breeding programmes.

Molecular-based biological and geographical diversity differed with respect to allelic richness, frequency of rare alleles, the common and most frequent alleles, and group-specific unique alleles. The differences in sample size of the germplasm included in each group may partially explain these differences. However, we also detected differences in mean molecular weight for the amplified fragments (allele size range) produced by different groups. For example, the average allele-size range of the desi types (113.9 bp) was greater by 12.2 bp than kabuli types (101.7 bp); pea-shaped allele-size range (55.4 bp) lower by 58.5 bp from desi and by 46.3 bp from kabuli types; the Mediterranean and SSEA accessions had similar average allele-size range (103.5 bp to 104.8 bp) while African accessions showed much reduced allele size range (68.1 bp) that differ by 32–37 bp from those of Mediterranean, SSEA, and WA region accessions (100.6 bp to 104.8 bp). The reduced allele size range observed in pea-shaped types or with those from African accessions could probably be due to founder effects (population size) associated with chickpea evolution and domestication [4], SSR evolution [31-33,35], or dilution of genetic variation as the pea-shaped most probably originated as a result of introgression between desi and kabuli types.

Reduced marker polymorphisms, as measured by differences in PIC values between groups of accessions, were detected in the reference set in comparison with composite collection. However, few markers in desi, kabuli, and pea-shaped among biological types and Africa and Mediterranean region accessions among geographical types were more polymorphic in the reference set than in composite collection. Both Mediterranean and Africa regions, respectively, are the center of origin [38] and secondary center of diversity [39] of chickpea, thus genetically more diverse than other region accessions. The highly polymorphic markers and genetically diverse germplasm with beneficial traits from the Mediterranean and Africa region accessions are probably the best source materials for use in chickpea genomics and breeding.

Neighbour-joining tree broadly separated kabuli from desi types, with pea-shaped types dispersed in both the groups, and wild *Cicer *accessions falling within kabuli types. However, *C. echinospermum *separated from *C. reticulatum*, though both belong to the primary gene pool and are cross compatible with *C. arietinum *(Figure 1). A reference set of 300 most diverse accessions has been formed that captured 1315 (78%) of the 1683 alleles, representing diversity from the entire spectrum of composite collection. From preliminary evaluation of this reference set for various agronomic traits at Patancheru, India, a number of accessions with beneficial traits were identified: 18 tolerant to drought, 12 to salinity, 4 to pod borer, 55 to dry root rot, 21 to fusarium wilt, and 3 to ascohcyhta blight, while 4 to 5 accessions each with variation in early maturity, large-seed size, high seed yield and high protein content (ICRISAT unpublished data). This reference set is therefore a useful resource for identifying diverse lines for use in functional and comparative genomics, in mapping and cloning gene(s), and in applied plant breeding for enhancing the genetic potential of chickpea. Further work is in progress at ICRISAT to add more number of markers and phenotype to this reference set for agronomic traits including drought, salinity and high temperature tolerance. Limited seed stock of this reference set is available upon request to researchers after signing Standard Material Transfer Agreement .

## Conclusion

Crop improvement depends on the existence of genetic diversity. We report here the largest ever study undertaken in chickpea to characterize genetic structure and allelic diversity, using SSR (48) in high throughput assay (ABI3700 and ABI3100), in composite collection (3000 accessions), and formation of a genotype-based reference set (300 accessions). This reference set captured 1315 of the 1683 (78%) alleles, representing diversity from all direction of the Neighbour-joining tree diagram, of the composite collection. It is a useful resource for allele mining, association genetics, mapping and cloning of gene(s), and in applied breeding to broaden the genetic base of chickpea.

## Methods

### Plant material and DNA extraction

All the 3000 accessions of the chickpea composite collection  including the two internal controls, Annigeri (ICC 4918) and ICCV 2, were grown in the field. ICCV 2 is an early maturing (flowers about two weeks earlier and matures one week earlier than Annigeri) kabuli chickpea with resistance to wilt (*Fusarium oxysporum *f. sp. *ciceri *race 1) [40], and released for cultivation in India (as Swetha), Sudan (as Wad Hamid) and Myanmar (as Yezin 3) [41]. Annigeri belongs to desi chickpea and was released for its earliness and wide adaptation for cultivation in the peninsular India [42]. A single plant from each accession was harvested and the seeds obtained from such plants were used to raise seedlings for DNA extraction. Young leaf tissues of each accession from the greenhouse grown plants were harvested and immediately stored in 96-well plate that consists of 94 accessions and two controls (Annigeri and ICCV 2). The two controls were added to each set of 94 accessions placed in 96-well plates for DNA extraction. DNA isolation for all 3000 accessions was carried out at ICRISAT.

A high-throughput DNA isolation protocol [43] was adopted to isolate DNA from the leaf tissues in 96-well format. DNA quantification, quality check and normalization to 5 ng/μl were done on agarose gel (0.8%) using lambda DNA standard (MBI Fermentas, USA). DNA isolated for all the 3000 accessions at ICRISAT was supplied to ICARDA for genotyping with 15 SSR markers.

### Identification of polymorphic SSR markers

From the preliminary screening of 200 SSR markers on a chickpea mini core collection of 211 accessions [7], 50 polymorphic SSR markers were selected to genotype the composite collection [19-21]. Of these, six SSRs belong to dinucleotide repeats, 35 to trinucleotide repeats, and the remaining nine to compound repeats. Thirty-seven of the 50 SSRs mapped on chickpea genome [20,24], representing 3 to 9 SSR loci on each of the eight chromosomes (see additional file 5).

### Polymerase chain reaction (PCR) and genotyping

Genotyping of the composite collection was performed in two labs. ICRISAT generated data for 35 SSR loci on 3000 accessions using an ABI3700 Genetic Analyzer (Applied Biosystems, USA), while ICARDA generated data for 15 SSR on 3000 accessions using an ABI3100 Genetic Analyzer (Applied Biosystems, USA).

PCR reactions were performed in 5 μl volumes in either 384-well PCR plates (ABGene, Rochester, N.Y.) or 96-well plates. Each PCR reaction contained 5 ng of genomic DNA, 2–4 pmol of primers, 1–4 mM MgCl_2_, 0.1–0.2 mM dNTP, 0.4 U of Qiagen *Taq *polymerase (Applied Biosystems) and 1× PCR buffer (Applied Biosystems). PCR amplification was carried out using touch down methodology with 3 minutes initial denaturation, followed by 5 cycles of 94°C for 20 seconds, 60°C for 20 seconds and 72°C for 30 seconds, then by 30 cycles of 94°C for 10 seconds, appropriate annealing temp for 20 seconds, and 72°C for 30 seconds. After completion of all 35 cycles, a final extension of 20 min at 72°C was performed. For amplification of some of the loci, PCR cycles were programmed for 2 min initial denaturation at 94°C, followed by 35 cycles of 20 s at 94°C, 50 s at 55°C and at 50 s at 72°C; and followed with a final extension of 5 min at 72°C.

PCR products generated by four different fluorescence dye-labeled primers were pooled in equal volumes and 1.5 μl each of FAM- VIC- NED- and PET-labeled product were mixed with 7 μl of formamide (Applied Biosystems), 0.25 μl of the GeneScan™ 500 LIZ^® ^Size Standard (Applied Biosystems) and 2.75 μl of distilled water. DNA fragments were denatured and size fractioned using capillary electrophoresis on an ABI 3700 or ABI 3100 DNA Genetic Analyzer (Applied Biosystems, USA). Whenever GeneScan™ 500 ROX^® ^Size Standard (Applied Biosystems) was used, equal amount of FAM, NED and VIC labeled PCR products were mixed and denatured as above and size fractioned using capillary electrophoresis on an ABI 3100 Genetic Analyzer. Subsequently, the Genscan 3.1 software (PE- Applied Biosystems) was applied to size peak patterns, using the internal LIZ-500 size standard and Genotyper 3.1 (PE- Applied Biosystems) was used for allele calling. At ICARDA, for the estimation of allele sizes of the 15 SSR markers, GeneMapper v3.5 (Applied Biosystems) was used.

### Data analysis

Accessions with more than 5% missing data were dropped from the analysis, thus, only 48 SSR loci data on 2915 accessions were used for statistical analysis. Called allelic data were used to determine the accurate size of the allele, tested against its standard deviation (*S*_*w*_) using the AlleloBin programme [44]. The fragments are first sorted in descending order by size, those with less than 0.4 bp are binned together (*S*_*w *_of each bin below 0.2 bp), and the mean is determined and rounded off to the nearest whole base-pair integer to give a molecular weight of the allele. *S*_*w *_provides the measure of accuracy of binning/allele size: ≤ 0.30 accurate allele size; 0.31–0.40 allele size likely to be good, 0.41–0.45 poor allele size, and > 0.45 unacceptable allele size. All the markers, except TA28 (*S*_*w *_= 0.536), showed the accepted allele size (data not presented). Further, TR2 showed high heterozygosity (79.52%), most likely due to a duplicate locus. These two markers (TA28 and TR2) were dropped and only data set of 48 SSR loci on 2915 accessions (with less than 3.25% missing data) of the composite collection was used for statistical analysis.

The basic statistics such as polymorphic information content (PIC), allelic richness as determined by a total number of the detected alleles and a number of alleles per locus, gene diversity, and occurrence of unique, rare, common, and most frequent alleles, and heterozygosity (%) were estimated using the PowerMarker V3.0 [[45], ]. Unique alleles are those that are present in one accession or one group of accessions but absent in other accessions or group of accessions. Rare alleles are those whose frequency is ≤ 1% in the investigated materials. Common alleles are those occurring between 1%–20% in the investigated materials while those occurring with > 20% classified as most frequent alleles.

Simple matching allele frequency-based distance matrix was used in DARwin-5.0 program [46] to dissect the genetic structure of the composite collection (2915 accessions and 48 SSR loci). We used "maximum length sub tree" method in DARwin 5.0 to select a reference set of 300 most diverse accessions. This procedure allows the choice of the sample size to retain the diversity, which is expressed by the tree as build on the initial set of accessions (2915 accessions in this case). The two accessions are redundant if the distance in the tree, as judged by the length of edges, is small. The accessions with longest edge have more uncommon characters and therefore genetically most diverse. For a particular sample size, the composition and the corresponding sub tree can be recorded.

## Authors' contributions

HDU, SLD, MB, and SMU contributed equally in conceiving the study and developing composite collection; HDU and SLD responsible for growing composite collection accessions in greenhouse for collection of leaf samples for extracting DNA, and analyzing data and drafting the manuscript; SMU, MB, RKV, and DH were responsible for generation of marker data; RKV, MB, SMU, DH, and CLLG also contributed towards writing the manuscript; SS participated in development of composite collection and analyzed marker data using PowerMarker and DARwin-5.0 structure program. All authors read and approved the manuscript.

## Supplementary Material

Additional file 1 **Variation in allele size range as revealed from the biologically and geographically distinct chickpea accessions for 48 SSR loci.** Click here for file

Additional file 2 **PIC values for individual markers in desi, kabuli, and pea-shaped chickpea accessions included in composite collection and reference set.** Click here for file

Additional file 3 **PIC values for individual markers in region-specific chickpea accessions included in composite collection and reference set.** Click here for file

Additional file 4 **Country of origin and biological status of 300 accessions included in chickpea reference set.** Click here for file

Additional file 5 **Chickpea genetic map **[20,24]** with putative position of the 37 of 48 SSR markers, in eight linkage groups, used in this study.** Click here for file
