# Supplementary material for: Genetic structure, diversity, and allelic richness in composite collection and reference set in chickpea (Cicer arietinum L.)
Source: BMC Plant Biol. 2008 Oct 16;8:106. doi: 10.1186/1471-2229-8-106 (PMC2583987; doi:10.1186/1471-2229-8-106)
Supplement: Additional file 1 — Variation in allele size range as revealed from the biologically and geographically distinct chickpea accessions for 48 SSR loci. [file 1471-2229-8-106-S1.doc]

**Additional file 1: Variation in allele size range as revealed from the biologically and geographically distinct chickpea accessions for 48 SSR loci**

| **SSR loci** | **Allele size range (bp)** | | | | | | | |
| --- | --- | --- | --- | --- | --- | --- | --- | --- |
|  | **Biological** | | | | **Geographical** | | | |
|  | Desi  (1668 accessions) | Kabuli  (1167 accessions) | Pea-shaped  (70 accessions) | Wild (10 accessions) | Africa  (215 accessions) | Mediterran-ean (619 accessions) | SSEA  (1138 accessions) | WA (720 accessions) |
| CaSTMS2 | 114 | 96 | 66 | 18 | 72 | 90 | 72 | 114 |
| CaSTMS15 | 144 | 120 | 60 | 105 | 51 | 129 | 144 | 114 |
| CaSTMS21 | 60 | 48 | 42 | 18 | 39 | 60 | 54 | 54 |
| NCPGR4 | 28 | 50 | 18 | 18 | 20 | 50 | 28 | 20 |
| NCPGR6 | 148 | 138 | 62 | 24 | 62 | 78 | 126 | 110 |
| NCPGR7 | 40 | 12 | 8 | 24 | 8 | 42 | 30 | 40 |
| NCPGR12 | 58 | 48 | 34 | 50 | 44 | 58 | 48 | 42 |
| NCPGR19 | 174 | 168 | 18 | 16 | 24 | 24 | 170 | 176 |
| TA2 | 158 | 104 | 67 | 39 | 106 | 107 | 125 | 143 |
| TA3 | 77 | 100 | 15 | 22 | 18 | 77 | 74 | 94 |
| TA5 | 132 | 120 | 75 | 51 | 99 | 120 | 126 | 114 |
| TA8 | 84 | 78 | 51 | 91 | 66 | 106 | 81 | 72 |
| TA11 | 81 | 66 | 39 | 108 | 51 | 111 | 51 | 72 |
| TA14 | 144 | 102 | 54 | 84 | 93 | 84 | 138 | 114 |
| TA21 | 138 | 147 | 75 | 48 | 96 | 114 | 102 | 141 |
| TA22 | 153 | 114 | 93 | 81 | 111 | 180 | 144 | 120 |
| TA27 | 90 | 69 | 42 | 51 | 72 | 84 | 90 | 69 |
| TA42 | 147 | 120 | 78 | 99 | 108 | 138 | 147 | 129 |
| TA46 | 69 | 57 | 45 | 3 | 54 | 66 | 66 | 66 |
| TA64 | 93 | 99 | 75 | 78 | 75 | 111 | 90 | 75 |
| TA71 | 123 | 135 | 72 | 72 | 84 | 135 | 123 | 108 |
| TA72 | 198 | 180 | 33 | 54 | 66 | 147 | 192 | 195 |
| TA76s | 93 | 132 | 54 | 102 | 69 | 165 | 111 | 96 |
| TA78 | 167 | 140 | 51 | 51 | 84 | 102 | 114 | 152 |
| TA80 | 127 | 97 | 45 | 48 | 94 | 54 | 124 | 97 |
| TA96 | 163 | 126 | 57 | 75 | 63 | 150 | 138 | 139 |
| TA113 | 69 | 54 | 24 | 69 | 36 | 69 | 54 | 69 |
| TA116 | 114 | 93 | 48 | 84 | 57 | 105 | 114 | 81 |
| TA117 | 126 | 141 | 45 | 66 | 69 | 102 | 93 | 141 |
| TA118 | 117 | 102 | 114 | 111 | 99 | 138 | 111 | 123 |
| TA130 | 66 | 63 | 48 | 66 | 63 | 69 | 66 | 57 |
| TA135 | 45 | 45 | 30 | 69 | 45 | 96 | 45 | 45 |
| TA142 | 48 | 75 | 42 | 84 | 30 | 84 | 45 | 48 |
| TA144 | 99 | 96 | 81 | 81 | 68 | 101 | 99 | 84 |
| TA176 | 189 | 198 | 138 | 126 | 138 | 201 | 183 | 204 |
| TA194 | 204 | 105 | 48 | 75 | 57 | 87 | 204 | 102 |
| TA200 | 111 | 129 | 57 | 57 | 90 | 123 | 96 | 114 |
| TA203 | 134 | 122 | 110 | 27 | 106 | 122 | 119 | 134 |
| TA206 | 99 | 90 | 54 | 39 | 42 | 93 | 90 | 99 |
| TAA58 | 126 | 117 | 60 | 87 | 99 | 144 | 120 | 114 |
| TAASH | 123 | 117 | 63 | 0 | 102 | 108 | 102 | 123 |
| TR1 | 173 | 149 | 85 | 119 | 101 | 152 | 158 | 173 |
| TR7 | 72 | 63 | 57 | 48 | 60 | 75 | 72 | 63 |
| TR29 | 96 | 93 | 48 | 87 | 51 | 120 | 96 | 81 |
| TR31 | 66 | 30 | 24 | 42 | 24 | 42 | 66 | 39 |
| TR43 | 177 | 186 | 120 | 96 | 135 | 198 | 180 | 123 |
| TS45 | 140 | 102 | 24 | 105 | 36 | 114 | 140 | 102 |
| TS84 | 69 | 45 | 12 | 39 | 33 | 45 | 69 | 45 |
| Mean (bp) | 113.9 | 101.7 | 55.4 | 62.6 | 68.1 | 103.5 | 104.8 | 100.6 |

SSEA = South and Southeast Asia; WA = West Asia
