# Supplementary material for: Genetic structure, diversity, and allelic richness in composite collection and reference set in chickpea (Cicer arietinum L.)
Source: BMC Plant Biol. 2008 Oct 16;8:106. doi: 10.1186/1471-2229-8-106 (PMC2583987; doi:10.1186/1471-2229-8-106)
Supplement: Additional file 2 — PIC values for individual markers in desi, kabuli, and pea-shaped chickpea accessions included in composite collection and reference set. [file 1471-2229-8-106-S2.doc]

**Additional file 2:** **PIC values for individual markers in desi, kabuli, and pea-shaped chickpea accessions included in composite collection and reference set**

|  | **Desi** |  | **Kabuli** |  | **Pea-shaped** |  |
| --- | --- | --- | --- | --- | --- | --- |
| **Marker** | Composite | Reference | Composite | Reference | Composite | Reference |
| CaSTMS2 | 0.931 | 0.928 | 0.908 | 0.917 | 0.890 | 0.820 |
| CaSTMS15 | 0.895 | 0.903 | 0.905 | 0.911 | 0.892 | 0.766 |
| CaSTMS21 | 0.379 | 0.399 | 0.514 | 0.591 | 0.556 | 0.466 |
| NCPGR4 | 0.704 | 0.684 | 0.244 | 0.321 | 0.447 | 0.450 |
| NCPGR6 | 0.448 | 0.527 | 0.530 | 0.577 | 0.601 | 0.663 |
| NCPGR7 | 0.448 | 0.499 | 0.511 | 0.564 | 0.586 | 0.563 |
| NCPGR12 | 0.781 | 0.803 | 0.796 | 0.831 | 0.803 | 0.642 |
| NCPGR19 | 0.467 | 0.515 | 0.556 | 0.599 | 0.566 | 0.640 |
| TA2 | 0.940 | 0.947 | 0.931 | 0.953 | 0.913 | 0.849 |
| TA3 | 0.646 | 0.649 | 0.647 | 0.734 | 0.678 | 0.645 |
| TA5 | 0.899 | 0.908 | 0.892 | 0.897 | 0.836 | 0.701 |
| TA8 | 0.913 | 0.930 | 0.903 | 0.913 | 0.878 | 0.844 |
| TA11 | 0.831 | 0.848 | 0.878 | 0.886 | 0.859 | 0.772 |
| TA14 | 0.906 | 0.918 | 0.869 | 0.886 | 0.862 | 0.798 |
| TA21 | 0.926 | 0.926 | 0.948 | 0.944 | 0.911 | 0.844 |
| TA22 | 0.952 | 0.951 | 0.920 | 0.933 | 0.910 | 0.849 |
| TA27 | 0.876 | 0.875 | 0.870 | 0.852 | 0.857 | 0.820 |
| TA42 | 0.921 | 0.933 | 0.934 | 0.936 | 0.883 | 0.868 |
| TA46 | 0.814 | 0.860 | 0.857 | 0.872 | 0.842 | 0.699 |
| TA64 | 0.945 | 0.943 | 0.932 | 0.928 | 0.923 | 0.820 |
| TA71 | 0.900 | 0.938 | 0.922 | 0.918 | 0.879 | 0.868 |
| TA72 | 0.880 | 0.893 | 0.856 | 0.871 | 0.841 | 0.819 |
| TA76s | 0.756 | 0.764 | 0.776 | 0.740 | 0.851 | 0.772 |
| TA78 | 0.867 | 0.891 | 0.923 | 0.942 | 0.846 | 0.772 |
| TA80 | 0.924 | 0.926 | 0.867 | 0.876 | 0.858 | 0.844 |
| TA96 | 0.892 | 0.898 | 0.914 | 0.892 | 0.896 | 0.868 |
| TA113 | 0.818 | 0.857 | 0.846 | 0.855 | 0.792 | 0.820 |
| TA116 | 0.815 | 0.838 | 0.823 | 0.869 | 0.761 | 0.672 |
| TA117 | 0.922 | 0.928 | 0.935 | 0.940 | 0.907 | 0.844 |
| TA118 | 0.918 | 0.925 | 0.954 | 0.942 | 0.936 | 0.858 |
| TA130 | 0.803 | 0.822 | 0.815 | 0.845 | 0.713 | 0.640 |
| TA135 | 0.821 | 0.843 | 0.860 | 0.875 | 0.634 | 0.410 |
| TA142 | 0.667 | 0.751 | 0.753 | 0.757 | 0.686 | 0.581 |
| TA144 | 0.943 | 0.940 | 0.846 | 0.918 | 0.897 | 0.868 |
| TA176 | 0.973 | 0.958 | 0.957 | 0.936 | 0.953 | 0.868 |
| TA194 | 0.811 | 0.828 | 0.880 | 0.889 | 0.864 | 0.891 |
| TA200 | 0.904 | 0.915 | 0.906 | 0.906 | 0.873 | 0.745 |
| TA203 | 0.964 | 0.962 | 0.942 | 0.912 | 0.934 | 0.844 |
| TA206 | 0.876 | 0.889 | 0.908 | 0.917 | 0.896 | 0.868 |
| TAA58 | 0.942 | 0.944 | 0.963 | 0.948 | 0.910 | 0.868 |
| TaaSH | 0.921 | 0.917 | 0.927 | 0.933 | 0.894 | 0.761 |
| TR1 | 0.920 | 0.908 | 0.920 | 0.909 | 0.859 | 0.798 |
| TR7 | 0.868 | 0.870 | 0.891 | 0.892 | 0.898 | 0.788 |
| TR29 | 0.907 | 0.912 | 0.914 | 0.918 | 0.889 | 0.844 |
| TR31 | 0.838 | 0.846 | 0.766 | 0.790 | 0.825 | 0.730 |
| TR43 | 0.949 | 0.954 | 0.922 | 0.895 | 0.915 | 0.820 |
| TS45 | 0.855 | 0.860 | 0.814 | 0.843 | 0.816 | 0.798 |
| TS84 | 0.609 | 0.670 | 0.508 | 0.578 | 0.559 | 0.535 |
| Mean | 0.831 | 0.846 | 0.830 | 0.845 | 0.818 | 0.757 |
| Minimum | 0.379 | 0.399 | 0.244 | 0.321 | 0.447 | 0.410 |
| Maximum | 0.973 | 0.962 | 0.963 | 0.953 | 0.953 | 0.891 |
