# Supplementary material for: Genetic structure, diversity, and allelic richness in composite collection and reference set in chickpea (Cicer arietinum L.)
Source: BMC Plant Biol. 2008 Oct 16;8:106. doi: 10.1186/1471-2229-8-106 (PMC2583987; doi:10.1186/1471-2229-8-106)
Supplement: Additional file 3 — PIC values for individual markers in region-specific chickpea accessions included in composite collection and reference set. [file 1471-2229-8-106-S3.doc]

**Additional file 3:** **PIC values for individual markers in region-specific chickpea accessions included in composite collection and reference set**

|  | **Africa** | | **Mediterranean** | | **South and South East Asia (SSEA)** | | **West Asia (WA)** | |
| --- | --- | --- | --- | --- | --- | --- | --- | --- |
| Marker | Composite | Reference | Composite | Reference | Composite | Reference | Composite | Reference |
| CaSTMS2 | 0.927 | 0.909 | 0.911 | 0.923 | 0.918 | 0.910 | 0.933 | 0.931 |
| CaSTMS15 | 0.894 | 0.894 | 0.896 | 0.900 | 0.880 | 0.874 | 0.912 | 0.887 |
| CaSTMS21 | 0.322 | 0.237 | 0.629 | 0.720 | 0.321 | 0.257 | 0.491 | 0.544 |
| NCPGR4 | 0.290 | 0.407 | 0.260 | 0.524 | 0.685 | 0.655 | 0.452 | 0.457 |
| NCPGR6 | 0.402 | 0.556 | 0.467 | 0.581 | 0.390 | 0.431 | 0.488 | 0.534 |
| NCPGR7 | 0.349 | 0.509 | 0.453 | 0.526 | 0.383 | 0.435 | 0.503 | 0.514 |
| NCPGR12 | 0.401 | 0.508 | 0.727 | 0.843 | 0.736 | 0.725 | 0.827 | 0.826 |
| NCPGR19 | 0.357 | 0.481 | 0.611 | 0.745 | 0.447 | 0.440 | 0.604 | 0.614 |
| TA2 | 0.920 | 0.879 | 0.914 | 0.936 | 0.925 | 0.925 | 0.947 | 0.950 |
| TA3 | 0.710 | 0.647 | 0.677 | 0.794 | 0.547 | 0.462 | 0.766 | 0.784 |
| TA5 | 0.841 | 0.836 | 0.891 | 0.917 | 0.886 | 0.892 | 0.915 | 0.912 |
| TA8 | 0.922 | 0.910 | 0.919 | 0.925 | 0.905 | 0.912 | 0.905 | 0.919 |
| TA11 | 0.790 | 0.674 | 0.857 | 0.908 | 0.788 | 0.792 | 0.889 | 0.894 |
| TA14 | 0.866 | 0.846 | 0.857 | 0.896 | 0.892 | 0.899 | 0.930 | 0.927 |
| TA21 | 0.936 | 0.879 | 0.947 | 0.935 | 0.924 | 0.910 | 0.940 | 0.942 |
| TA22 | 0.947 | 0.904 | 0.896 | 0.935 | 0.947 | 0.923 | 0.956 | 0.950 |
| TA27 | 0.902 | 0.884 | 0.879 | 0.890 | 0.857 | 0.840 | 0.876 | 0.868 |
| TA42 | 0.894 | 0.910 | 0.928 | 0.931 | 0.908 | 0.925 | 0.949 | 0.942 |
| TA46 | 0.768 | 0.779 | 0.859 | 0.862 | 0.818 | 0.853 | 0.814 | 0.868 |
| TA64 | 0.923 | 0.879 | 0.935 | 0.938 | 0.945 | 0.940 | 0.939 | 0.929 |
| TA71 | 0.868 | 0.868 | 0.902 | 0.921 | 0.893 | 0.929 | 0.903 | 0.934 |
| TA72 | 0.862 | 0.825 | 0.854 | 0.872 | 0.883 | 0.879 | 0.860 | 0.882 |
| TA76s | 0.697 | 0.648 | 0.813 | 0.857 | 0.710 | 0.649 | 0.821 | 0.811 |
| TA78 | 0.855 | 0.793 | 0.928 | 0.906 | 0.861 | 0.878 | 0.911 | 0.916 |
| TA80 | 0.855 | 0.873 | 0.877 | 0.894 | 0.916 | 0.898 | 0.930 | 0.921 |
| TA96 | 0.912 | 0.879 | 0.906 | 0.891 | 0.863 | 0.852 | 0.936 | 0.937 |
| TA113 | 0.871 | 0.857 | 0.846 | 0.841 | 0.773 | 0.780 | 0.872 | 0.881 |
| TA116 | 0.720 | 0.715 | 0.840 | 0.877 | 0.756 | 0.714 | 0.873 | 0.899 |
| TA117 | 0.927 | 0.900 | 0.933 | 0.934 | 0.922 | 0.916 | 0.933 | 0.940 |
| TA118 | 0.913 | 0.896 | 0.952 | 0.948 | 0.913 | 0.903 | 0.948 | 0.944 |
| TA130 | 0.815 | 0.807 | 0.802 | 0.884 | 0.711 | 0.705 | 0.868 | 0.870 |
| TA135 | 0.743 | 0.757 | 0.877 | 0.904 | 0.768 | 0.762 | 0.851 | 0.863 |
| TA142 | 0.657 | 0.761 | 0.691 | 0.783 | 0.524 | 0.544 | 0.840 | 0.864 |
| TA144 | 0.908 | 0.895 | 0.813 | 0.929 | 0.936 | 0.913 | 0.924 | 0.940 |
| TA176 | 0.935 | 0.804 | 0.963 | 0.951 | 0.968 | 0.959 | 0.965 | 0.951 |
| TA194 | 0.781 | 0.796 | 0.868 | 0.868 | 0.781 | 0.826 | 0.817 | 0.791 |
| TA200 | 0.938 | 0.899 | 0.904 | 0.914 | 0.873 | 0.869 | 0.931 | 0.929 |
| TA203 | 0.945 | 0.910 | 0.941 | 0.927 | 0.960 | 0.953 | 0.967 | 0.960 |
| TA206 | 0.851 | 0.850 | 0.918 | 0.921 | 0.860 | 0.870 | 0.886 | 0.877 |
| TAA58 | 0.912 | 0.889 | 0.965 | 0.952 | 0.936 | 0.929 | 0.953 | 0.950 |
| TAASH | 0.919 | 0.882 | 0.932 | 0.941 | 0.910 | 0.901 | 0.932 | 0.915 |
| TR1 | 0.872 | 0.889 | 0.920 | 0.909 | 0.908 | 0.892 | 0.943 | 0.925 |
| TR7 | 0.813 | 0.802 | 0.883 | 0.874 | 0.861 | 0.844 | 0.913 | 0.895 |
| TR29 | 0.877 | 0.880 | 0.920 | 0.929 | 0.885 | 0.873 | 0.926 | 0.915 |
| TR31 | 0.661 | 0.671 | 0.801 | 0.822 | 0.807 | 0.822 | 0.793 | 0.795 |
| TR43 | 0.935 | 0.919 | 0.929 | 0.928 | 0.942 | 0.937 | 0.952 | 0.944 |
| TS45 | 0.796 | 0.796 | 0.821 | 0.879 | 0.831 | 0.857 | 0.870 | 0.855 |
| TS84 | 0.476 | 0.464 | 0.613 | 0.745 | 0.556 | 0.540 | 0.615 | 0.690 |
| Mean | 0.785 | 0.780 | 0.832 | 0.867 | 0.804 | 0.800 | 0.853 | 0.858 |
| Minimum | 0.290 | 0.237 | 0.260 | 0.524 | 0.321 | 0.257 | .452 | 0.457 |
| Maximum | 0.947 | 0.919 | 0.965 | 0.952 | 0.968 | 0.959 | 0.967 | 0.960 |
