# Supplementary material for: Genetic structure, diversity, and allelic richness in composite collection and reference set in chickpea (Cicer arietinum L.)
Source: BMC Plant Biol. 2008 Oct 16;8:106. doi: 10.1186/1471-2229-8-106 (PMC2583987; doi:10.1186/1471-2229-8-106)
Supplement: Additional file 4 — Country of origin and biological status of 300 accessions included in chickpea reference set. [file 1471-2229-8-106-S4.doc]

**Additional file 4: Country of origin and biological status of 300 accessions included in chickpea reference set**

| **Identity** | **Origin** | **Biological status** |
| --- | --- | --- |
| **Desi chickpea** | | |
| ICC 67 | India | Landrace |
| ICC 95 | India | Landrace |
| ICC 283 | India | Landrace |
| ICC 440 | India | Landrace |
| ICC 456 | India | Landrace |
| ICC 506 | India | Landrace |
| ICC 637 | India | Landrace |
| ICC 708 | India | Landrace |
| ICC 762 | India | Landrace |
| ICC 791 | India | Landrace |
| ICC 867 | India | Landrace |
| ICC 1052 | Pakistan | Landrace |
| ICC 1083 | Iran | Landrace |
| ICC 1098 | Iran | Landrace |
| ICC 1161 | Pakistan | Landrace |
| ICC 1164 | Nigeria | Landrace |
| ICC 1180 | India | Landrace |
| ICC 1194 | India | Landrace |
| ICC 1205 | India | Landrace |
| ICC 1230 | India | Landrace |
| ICC 1356 | India | Landrace |
| ICC 1392 | India | Landrace |
| ICC 1397 | India | Landrace |
| ICC 1398 | India | Landrace |
| ICC 1422 | India | Landrace |
| ICC 1431 | India | Landrace |
| ICC 1510 | India | Landrace |
| ICC 1710 | India | Landrace |
| ICC 1715 | India | Landrace |
| ICC 1882 | India | Landrace |
| ICC 1915 | India | Landrace |
| ICC 1923 | India | Landrace |
| ICC 2065 | India | Landrace |
| ICC 2072 | India | Landrace |
| ICC 2210 | Algeria | Landrace |
| ICC 2242 | India | Landrace |
| ICC 2263 | Iran | Landrace |
| ICC 2507 | Iran | Landrace |
| ICC 2580 | Iran | Landrace |
| ICC 2629 | Iran | Landrace |
| ICC 2679 | Iran | Landrace |
| ICC 2720 | Iran | Landrace |
| ICC 2737 | Iran | Landrace |
| ICC 2884 | Iran | Landrace |
| ICC 2919 | Iran | Landrace |
| ICC 2969 | Iran | Landrace |
| ICC 2990 | Iran | Landrace |
| ICC 3218 | Iran | Landrace |
| ICC 3230 | Iran | Landrace |
| ICC 3239 | Iran | Landrace |
| ICC 3325 | Cyprus | Landrace |
| ICC 3362 | Iran | Landrace |
| ICC 3391 | Iran | Landrace |
| ICC 3512 | Iran | Landrace |
| ICC 3582 | Iran | Landrace |
| ICC 3631 | Iran | Landrace |
| ICC 3761 | Iran | Landrace |
| ICC 3776 | Iran | Landrace |
| ICC 3892 | Iran | Landrace |
| ICC 3946 | Iran | Landrace |
| ICC 4093 | Iran | Landrace |
| ICC 4182 | Iran | Landrace |
| ICC 4363 | Iran | Landrace |
| ICC 4418 | Iran | Landrace |
| ICC 4463 | Iran | Landrace |
| ICC 4495 | Turkey | Landrace |
| ICC 4533 | India | Landrace |
| ICC 4567 | India | Landrace |
| ICC 4593 | India | Landrace |
| ICC 4639 | India | Landrace |
| ICC 4657 | India | Landrace |
| ICC 4814 | Iran | Landrace |
| ICC 4918 | India | Advanced cultivar |
| ICC 4991 | India | Advanced cultivar |
| ICC 5135 | India | Breeding material |
| ICC 5221 | India | Breeding material |
| ICC 5383 | India | Landrace |
| ICC 5434 | India | Landrace |
| ICC 5504 | Mexico | Landrace |
| ICC 5613 | India | Landrace |
| ICC 5639 | India | Landrace |
| ICC 5845 | India | Landrace |
| ICC 5878 | India | Landrace |
| ICC 6279 | India | Landrace |
| ICC 6293 | Italy | Landrace |
| ICC 6294 | Iran | Advanced cultivar |
| ICC 6306 | Union of Soviet Socialist Republics | Advanced cultivar |
| ICC 6537 | Iran | Breeding material |
| ICC 6571 | Iran | Landrace |
| ICC 6579 | Iran | Landrace |
| ICC 6802 | Iran | Landrace |
| ICC 6811 | Iran | Landrace |
| ICC 6816 | Iran | Landrace |
| ICC 6874 | Iran | Landrace |
| ICC 6875 | Iran | Landrace |
| ICC 6877 | Iran | Landrace |
| ICC 7052 | Iran | Landrace |
| ICC 7150 | Turkey | Landrace |
| ICC 7184 | Turkey | Landrace |
| ICC 7305 | Afghanistan | Landrace |
| ICC 7326 | Unknown | Landrace |
| ICC 7441 | India | Landrace |
| ICC 7554 | Iran | Landrace |
| ICC 7819 | Iran | Landrace |
| ICC 7867 | Iran | Landrace |
| ICC 8195 | Pakistan | Landrace |
| ICC 8200 | Iran | Landrace |
| ICC 8318 | India | Landrace |
| ICC 8384 | India | Landrace |
| ICC 8515 | Greece | Landrace |
| ICC 8521 | Italy | Landrace |
| ICC 8522 | Italy | Landrace |
| ICC 8607 | Ethiopia | Landrace |
| ICC 8621 | Ethiopia | Landrace |
| ICC 8718 | Afghanistan | Landrace |
| ICC 8950 | India | Landrace |
| ICC 9002 | Iran | Landrace |
| ICC 9586 | India | Landrace |
| ICC 9590 | Egypt | Landrace |
| ICC 9636 | Afghanistan | Landrace |
| ICC 9643 | Afghanistan | Landrace |
| ICC 9702 | Afghanistan | Landrace |
| ICC 9712 | Afghanistan | Landrace |
| ICC 9755 | Afghanistan | Landrace |
| ICC 9942 | India | Landrace |
| ICC 10018 | India | Landrace |
| ICC 10393 | India | Landrace |
| ICC 10399 | India | Landrace |
| ICC 10673 | Turkey | Landrace |
| ICC 10685 | Turkey | Landrace |
| ICC 10939 | India | Landrace |
| ICC 10945 | India | Landrace |
| ICC 11121 | India | Landrace |
| ICC 11198 | India | Landrace |
| ICC 11279 | Pakistan | Landrace |
| ICC 11284 | Union of Soviet Socialist Republics | Landrace |
| ICC 11378 | India | Landrace |
| ICC 11498 | India | Breeding material |
| ICC 11584 | India | Landrace |
| ICC 11627 | India | Landrace |
| ICC 11664 | India | Landrace |
| ICC 11903 | Germany | Landrace |
| ICC 11944 | Nepal | Landrace |
| ICC 12028 | Mexico | Landrace |
| ICC 12155 | Bangladesh | Landrace |
| ICC 12299 | Nepal | Landrace |
| ICC 12307 | Myanmar | Landrace |
| ICC 12321 | Unknown | Landrace |
| ICC 12379 | Iran | Landrace |
| ICC 12537 | Ethiopia | Landrace |
| ICC 12654 | Ethiopia | Landrace |
| ICC 12726 | Ethiopia | Landrace |
| ICC 12824 | Ethiopia | Landrace |
| ICC 12851 | Ethiopia | Landrace |
| ICC 12866 | Ethiopia | Landrace |
| ICC 12916 | India | Landrace |
| ICC 12928 | India | Landrace |
| ICC 12947 | India | Landrace |
| ICC 13124 | India | Landrace |
| ICC 13219 | Iran | Landrace |
| ICC 13524 | Iran | Landrace |
| ICC 13599 | Iran | Landrace |
| ICC 13863 | Ethiopia | Landrace |
| ICC 13892 | Ethiopia | Landrace |
| ICC 14051 | Ethiopia | Landrace |
| ICC 14077 | Ethiopia | Landrace |
| ICC 14098 | Ethiopia | Landrace |
| ICC 14402 | India | Breeding material |
| ICC 14595 | India | Landrace |
| ICC 14669 | India | Landrace |
| ICC 14778 | India | Landrace |
| ICC 14799 | India | Landrace |
| ICC 14815 | India | Landrace |
| ICC 14831 | India | Landrace |
| ICC 15248 | Iran | Landrace |
| ICC 15294 | Iran | Landrace |
| ICC 15510 | Morocco | Landrace |
| ICC 15567 | India | Breeding material |
| ICC 15606 | India | Landrace |
| ICC 15610 | India | Landrace |
| ICC 15612 | Tanzania | Landrace |
| ICC 15614 | Tanzania | Landrace |
| ICC 15618 | India | Landrace |
| ICC 15762 | Syria | Landrace |
| ICC 15785 | Syria | Landrace |
| ICC 15868 | India | Landrace |
| ICC 16207 | Myanmar | Landrace |
| ICC 16261 | Malawi | Landrace |
| ICC 16269 | Malawi | Landrace |
| ICC 16374 | Malawi | Breeding material |
| ICC 16487 | Pakistan | Landrace |
| ICC 16524 | Pakistan | Landrace |
| ICC 16903 | India | Landrace |
| ICC 16915 | India | Landrace |
| **Kabuli chickpea** | | |
| ICC 2277 | Iran | Landrace |
| ICC 2482 | Iran | Landrace |
| ICC 2593 | Iran | Landrace |
| ICC 3410 | Iran | Landrace |
| ICC 3421 | Israel | Landrace |
| ICC 4841 | Morocco | Landrace |
| ICC 4853 | Unknown | Landrace |
| ICC 5337 | India | Landrace |
| ICC 6263 | Union of Soviet Socialist Republics | Landrace |
| ICC 7255 | India | Landrace |
| ICC 7272 | Algeria | Landrace |
| ICC 7308 | Peru | Landrace |
| ICC 7315 | Iran | Landrace |
| ICC 7571 | Isreal | Landrace |
| ICC 7668 | Union of Soviet Socialist Republics | Landrace |
| ICC 8058 | Iran | Landrace |
| ICC 8151 | USA | Landrace |
| ICC 8261 | Turkey | Landrace |
| ICC 8740 | Afghanistan | Landrace |
| ICC 8752 | Afghanistan | Landrace |
| ICC 8855 | Afghanistan | Landrace |
| ICC 9137 | Iran | Landrace |
| ICC 9402 | Iran | Landrace |
| ICC 9418 | Iran | Landrace |
| ICC 9434 | Iran | Landrace |
| ICC 9872 | Afghanistan | Landrace |
| ICC 10466 | India | Landrace |
| ICC 10755 | Turkey | Landrace |
| ICC 10885 | Ethiopia | Landrace |
| ICC 11303 | Chile | Landrace |
| ICC 11764 | Chile | Landrace |
| ICC 11819 | Chile | Landrace |
| ICC 11879 | Turkey | Landrace |
| ICC 12037 | Mexico | Breeding material |
| ICC 12324 | Unknown | Advanced cultivar |
| ICC 12328 | Cyprus | Landrace |
| ICC 12492 | India | Landrace |
| ICC 13077 | India | Landrace |
| ICC 13187 | Iran | Breeding material |
| ICC 13283 | Iran | Landrace |
| ICC 13357 | Iran | Landrace |
| ICC 13441 | Iran | Landrace |
| ICC 13461 | Iran | Landrace |
| ICC 13523 | Iran | Landrace |
| ICC 13628 | Iran | Landrace |
| ICC 13719 | Iran | Landrace |
| ICC 13764 | Iran | Landrace |
| ICC 13816 | Union of Soviet Socialist Republics | Landrace |
| ICC 14199 | Mexico | Breeding material |
| ICC 14446 | Italy | Landrace |
| ICC 15264 | Iran | Landrace |
| ICC 15333 | Iran | Landrace |
| ICC 15406 | Morocco | Landrace |
| ICC 15435 | Morocco | Landrace |
| ICC 15518 | Morocco | Landrace |
| ICC 15697 | Syria | Landrace |
| ICC 15802 | Syria | Landrace |
| ICC 16654 | China | Landrace |
| ICC 16796 | Portugal | Landrace |
| ICC 18679 | Iraq | Traditional cultivar/Landrace |
| ICC 18699 | Turkey | Traditional cultivar/Landrace |
| ICC 18720 | Morocco | Traditional cultivar/Landrace |
| ICC 18724 | Algeria | Traditional cultivar/Landrace |
| ICC 18828 | Syria | Traditional cultivar/Landrace |
| ICC 18836 | Syria | Unknown |
| ICC 18839 | Syria | Unknown |
| ICC 18847 | Syria | Unknown |
| ICC 18858 | Syria | Unknown |
| ICC 18884 | Syria | Unknown |
| ICC 18912 | Cyprus | Traditional cultivar/Landrace |
| ICC 18983 | Greece | Traditional cultivar/Landrace |
| ICC 19011 | Syria | Unknown |
| ICC 19034 | Uzbekistan | Traditional cultivar/Landrace |
| ICC 19095 | Turkey | Traditional cultivar/Landrace |
| ICC 19100 | Turkey | Traditional cultivar/Landrace |
| ICC 19122 | France | Unknown |
| ICC 19147 | Syria | Unknown |
| ICC 19165 | Italy | Unknown |
| IG 5949 | Unknown | Unknown |
| IG 6044 | Sudan | Traditional cultivar/Landrace |
| IG 6047 | Afghanistan | Traditional cultivar/Landrace |
| IG 6055 | Iran | Traditional cultivar/Landrace |
| IG 6067 | Turkey | Traditional cultivar/Landrace |
| IG 6154 | Iran | Traditional cultivar/Landrace |
| IG 7087 | Unknown | Unknown |
| IG 7296 | Afghanistan | Traditional cultivar/Landrace |
| ICCV 95311 | India | Breeding material |
| **Pea-shaped chickpea** | | |
| ICC 4872 | India | Landrace |
| ICC 5879 | India | Landrace |
| ICC 7323 | Union of Soviet Socialist Republics | Landrace |
| ICC 7413 | India | Landrace |
| ICC 8350 | India | Landrace |
| ICC 9848 | Afghanistan | Landrace |
| ICC 9862 | Afghanistan | Landrace |
| ICC 9895 | Afghanistan | Landrace |
| ICC 10341 | Turkey | Landrace |
| ICC 15888 | India | Landrace |
| ICC 19164 | Moldova | Unknown |
| ICC 19226 | France | Traditional cultivar/Landrace |
| **Wild *Cicer* species** | | |
| IG 69974 | Turkey | Cicer echinospermum |
| IG 72970 | Turkey | Cicer reticulatum |
| IG 73064 | Turkey | *Cicer echinospermum* |
| IG 73074 | Turkey | *Cicer echinospermum* |
| IG 73082 | Turkey | Cicer reticulatum |
| IG 73083 | Turkey | Cicer reticulatum |
| IG 73086 | Turkey | Cicer reticulatum |
