# Supplementary material for: Genetic structure, diversity, and allelic richness in composite collection and reference set in chickpea (Cicer arietinum L.)
Source: BMC Plant Biol. 2008 Oct 16;8:106. doi: 10.1186/1471-2229-8-106 (PMC2583987; doi:10.1186/1471-2229-8-106)
Supplement: Additional file 5 — Chickpea genetic map [20,24] with putative position of the 37 of 48 SSR markers, in eight linkage groups, used in this study. [file 1471-2229-8-106-S5.doc]

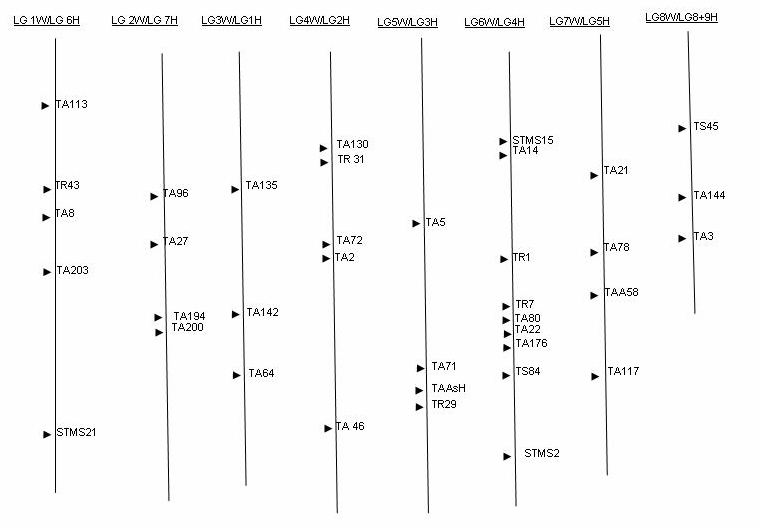


**Figure 3. Chickpea genetic map [20,24] with putative position of the 37 of 48 SSR markers, in eight linkage groups, used in this study**
